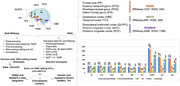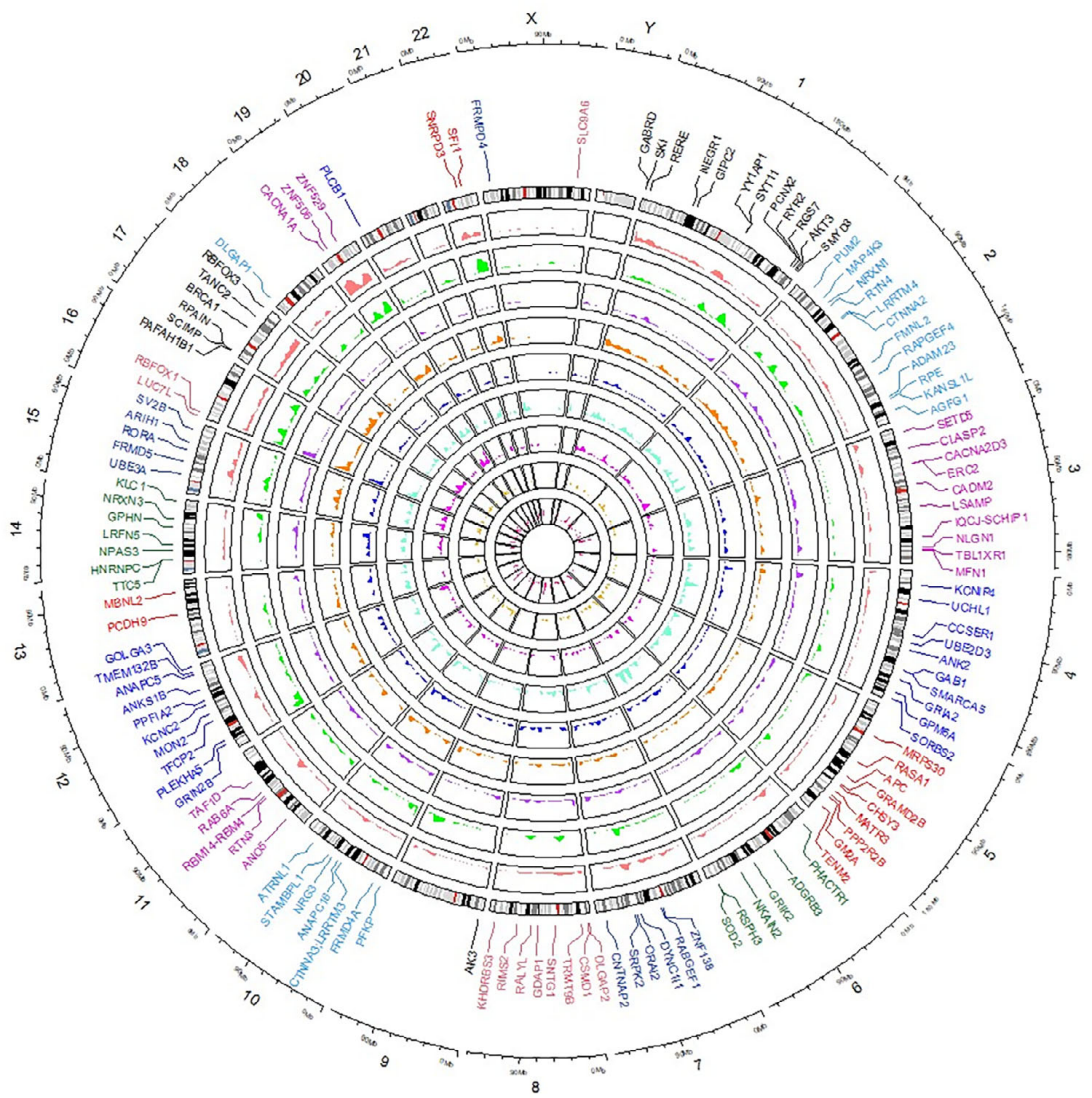# Genomic landscape of RNA editing and genome regulatory signatures in aged human brains with Alzheimer’s disease

**DOI:** 10.1002/alz.089503

**Published:** 2025-01-03

**Authors:** Amit Kumar Gupta, William Martin, Yayan Feng, Feixiong Cheng

**Affiliations:** ^1^ Cleveland Clinic, Cleveland, OH USA; ^2^ Case Western Reserve University, Cleveland, OH USA

## Abstract

**Background:**

RNA editing represents one of the most common post‐transcriptional modifications that contribute to transcriptomic diversity, impacting RNA stability and regulations. To this end, we sought to investigate brain region‐specific RNA‐editing signatures (RNA‐editings) associated with Alzheimer’s disease (AD) and the human aged brain with regulatory elements.

**Method:**

We investigated the genome‐wide landscape of RNA‐editings from 4,208 (1,364 AD case vs. 742 healthy controls [HC]) RNAseq samples across nine brain regions from three large biobanks (Mount Sinai, Mayo, ROSMAP) cohorts. We performed association and enrichment analysis of RNA‐editing enriched genes. We further identified sex‐specific and *APOE4*‐specific RNA‐editing. We inspected brain‐wide cis‐regulatory variants (RNA editing quantitative trait loci or cis‐edQTLs) considering two different cis‐distances (±100 KB and ±1 MB) utilizing matched whole‐genome genotyping data for 3,627 samples. Age, PMI, gender (sex), and *APOE4* status are used as adjusting covariates. GWAS and cis‐edQTLs (1 MB) colocalization were performed with downstream functional and genome regulatory analysis.

**Result:**

We have identified ∼14,687 significant RNA editing events across nine brain regions while comparing AD cases versus HC. We showed that two brain regions namely the parahippocampal gyrus (PHG), and cerebellum cortex (CBE) exhibit the highest editing abundance followed by the inferior frontal gyrus (IFG) and dorsolateral prefrontal cortex (DLPFC). Overall, we pinpointed 127 genes harboring significant RNA editing loci (5_*_10^‐3^) shared by two or more brain regions. Further, we uncovered a large repertoire (9650‐30386) of cis‐edQTLs (distance ±100 KB) among distinct brain regions potentially influencing RNA editing sites. A total of 76 colocalized GWAS and cis‐edQTL (±1 MB) genomic variants in about 43 genes were identified. Some of these genomic loci (genes + variants) are *CLU* (rs867230), *DGKQ* (rs4690197, rs3733347), *TRANK1* (rs7624498), *BIN1* (rs1060743), *PICALM* (rs639012), *FERMT2* (rs17125924), and *TSPAN14* (rs7098414). Functional enrichment analysis shows these genes are allied to synaptic cycles, amyloid‐beta formation, neurofibrillary tangle, immune pathways, AD disease pathways, protein metabolism and signal transduction.

**Conclusion:**

Our study offers brain‐wide region‐specific and common RNA editing signatures in AD. This underlying altered landscape of RNA‐editing and cis‐edQTLs may account for the genetic etiology and pathobiology of AD and other neurodegenerative diseases.